# An Unusual Cause of Diffuse Bone Pain in a Long‐Term Dialysis Patient: A Case Report of β2‐Microglobulin Amyloid Arthropathy

**DOI:** 10.1002/ccr3.72994

**Published:** 2026-06-19

**Authors:** Yannick Laurent Tchenadoyo Bayala, Mohamed Hanas Mama Sika, Marina Saley André Hamidou, Issa Ouedraogo, Rachidatou Kafando, Bakoubassé Aïssata Son, Wendyam Nadège Yameogo, Wendlassida Joëlle Stéphanie Zabsonré/Tiendrébéogo, Dieu‐Donné Ouedraogo

**Affiliations:** ^1^ Rheumatology Department Bogodogo University Hospital Center Ouagadougou Burkina Faso; ^2^ Nephrology Department Yalgado Ouedraogo University Hospital Center Ouagadougou Burkina Faso

**Keywords:** bone pain, destructive arthropathy, dialysis‐related amyloidosis, hemodialysis, β2‐microglobulin

## Abstract

Dialysis‐related amyloidosis (DRA) results from β2‐microglobulin accumulation in patients on long‐term hemodialysis, typically manifesting after 5–10 years. While well‐documented in developed countries, reports from sub‐Saharan Africa remain scarce. We report a 45 year‐old Burkinabe man with 8 years of maintenance hemodialysis who presented with severe diffuse bone pain and inflammatory polyarthralgia affecting shoulders, hips, knees, and wrists. Physical examination revealed diffuse spinal tenderness and restricted painful joint mobility with bilateral positive Patrick's and key signs. Laboratory investigations showed elevated C‐reactive protein and markedly elevated serum β2‐microglobulin, with negative rheumatoid factor, anti‐CCP antibodies, and antinuclear antibodies. MRI demonstrated characteristic band‐like vertebral infiltration with preserved disc spaces and extensive nodular involvement of the sacrum, iliac wings, and proximal femurs. Despite unavailable bone biopsy, probable DRA diagnosis was established based on prolonged low‐flux hemodialysis, markedly elevated β2‐microglobulin, characteristic imaging, and exclusion of alternative diagnoses including AL and AA amyloidosis. Treatment with celecoxib 200 mg daily achieved significant improvement at 1 month follow‐up. This case highlights probable DRA as an important cause of diffuse bone pain in younger African dialysis patients. Clinicians should maintain high suspicion for DRA in patients with polyarticular symptoms after long‐term dialysis, utilizing β2‐microglobulin measurement and MRI for diagnosis when histological confirmation is unavailable.

## Key Points

This case demonstrates that bone pain can be the presenting feature of probable DRA in patients after hemodialysis. The combination of markedly elevated serum β2‐microglobulin and characteristic MRI findings provides sufficient diagnostic certainty. Clinicians managing dialysis patients in resource‐limited settings should maintain high suspicion for DRA.

## 1 Introduction

Patients undergoing long‐term dialysis face multiple skeletal complications. While renal osteodystrophy remains the most recognized bone disorder in chronic kidney disease, dialysis‐related amyloidosis (DRA) represents an increasingly important cause of musculoskeletal morbidity in this population [1]. DRA results from the accumulation of β2‐microglobulin (β2‐m), a protein poorly cleared by conventional dialysis membranes [2]. Over time, typically after 5 to 10 years of dialysis therapy, elevated serum β2‐m levels lead to amyloid fibril deposition in osteoarticular tissues [3]. These deposits, modified by cytokines and macrophages, trigger local inflammation and progressive tissue destruction [3].

The clinical manifestations of DRA are well established and include carpal tunnel syndrome, destructive spondyloarthropathy, spinal stenosis, and bone cysts, particularly in the femoral head [1]. These complications significantly impact patients' quality of life and functional capacity. However, the pathophysiology of articular symptoms in DRA remains incompletely understood [4]. The relative contributions of direct mass effect from amyloid deposits, structural joint destruction, and inflammatory arthritis continue to be debated in the literature [4].

While DRA has been extensively documented in high‐income countries with established dialysis programs, reports from sub‐Saharan Africa remain limited. This gap in the literature partly reflects differences in dialysis access and survival rates. Younger patients on dialysis in resource‐limited settings may present diagnostic challenges, as DRA is less commonly considered in this demographic and geographic context [4].

We report a case of a 45 year‐old Burkinabe patient on long‐term hemodialysis who presented with diffuse bone pain. This case illustrates the clinical presentation of DRA in a relatively young African patient and highlights the importance of considering this diagnosis in the differential of bone pain in dialysis patients, regardless of geographic location or age.

## 2 Case Report

### 2.1 Patient Information

A 45 year‐old Burkinabe man presented to our rheumatology clinic with a 2 week exacerbation of diffuse bone pain. His medical history included hypertension diagnosed since 10 years, currently managed with amlodipine 10 mg daily. He had been on maintenance hemodialysis since 8 years for stage 5 chronic kidney disease, receiving two sessions per week through a functional left radiocephalic arteriovenous fistula. The use of low‐flux dialysis membranes, which inadequately clear β2‐microglobulin, likely contributed to the accumulation of this protein over his 8 years of dialysis treatment.

### 2.2 Clinical Findings

The patient reported a 2 year history of intermittent diffuse spinal pain and inflammatory polyarthralgia affecting large joints, shoulders, elbows, knees, hips, ankles, and wrists. These episodes initially followed a mixed inflammatory‐mechanical pattern but progressively became predominantly inflammatory. His current presentation consisted of severe spinal pain occurring at rest with mixed characteristics, associated with bilateral hip and shoulder pain that worsened toward the end of the day. Pain intensity was rated 8/10 on the visual analog scale. He denied fever, chills, fatigue, weight loss, or other constitutional symptoms. There were no cutaneous, visceral, or systemic manifestations, and no features suggestive of spondyloarthropathy.

Physical examination revealed tenderness along the entire spine without muscle spasm or stiffness. Spinal mobility was painful. Both hips and shoulders showed restricted and painful range of motion. Patrick's test was positive bilaterally. There were no signs of rotator cuff pathology. The key sign was positive bilaterally at the wrists. No myalgia was elicited.

### 2.3 Diagnostic Assessment

Laboratory investigations showed a white blood cell count of 6000/μL, normocytic normochromic anemia with hemoglobin at 8 g/dL, and platelets at 201,000/μL. C‐reactive protein was elevated at 26 mg/L. Estimated glomerular filtration rate was 12 mL/min/1.73m^2^. Serum β2‐microglobulin was markedly elevated at 52 mg/L (normal range: 1.0–2.4 mg/L), representing a 22‐fold increase above the upper limit of normal. Immunological workup including rheumatoid factor, anti‐cyclic citrullinated peptide antibodies, and antinuclear antibodies was negative. Serum electrolytes and liver function tests were within normal limits. Additional biochemical investigations relevant to bone pathology in dialysis patients showed: parathyroid hormone 125 pg/mL (normal range: 15–65 pg/mL), serum calcium 2.1 mmol/L (corrected, normal range: 2.2–2.6 mmol/L), serum phosphate 1.8 mmol/L (normal range: 0.8–1.5 mmol/L), and alkaline phosphatase 95 U/L (normal range: 30–120 U/L). Serum protein electrophoresis showed no monoclonal spike, and serum free light chain ratio (kappa/lambda) was 1.2 (normal range: 0.26–1.65), effectively excluding AL amyloidosis.

Thoracoabdominal pelvic CT scan revealed bilateral small kidneys with no visceral organ involvement. Magnetic resonance imaging of the spine and pelvis demonstrated characteristic findings, band‐like T1 and T2 hyperintense signal infiltration of multiple vertebral bodies with preserved intervertebral discs (Figures 1, 2, 3). In the pelvis, there was extensive nodular involvement of the sacrum, iliac wings, and proximal third of both femurs at the cervico‐trochanteric regions (Figures 4 and 5).

**FIGURE 1:**
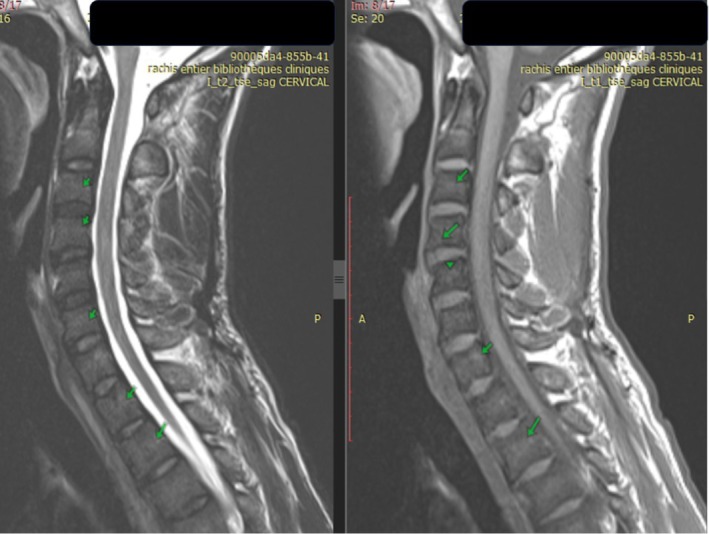
Sagittal T1 and T2‐weighted MRI of the cervical spine. Band‐like hyperintense signal infiltration of multiple cervical vertebral bodies (green arrows) with preserved intervertebral disc spaces, characteristic of β2‐microglobulin amyloid deposition.

**FIGURE 2:**
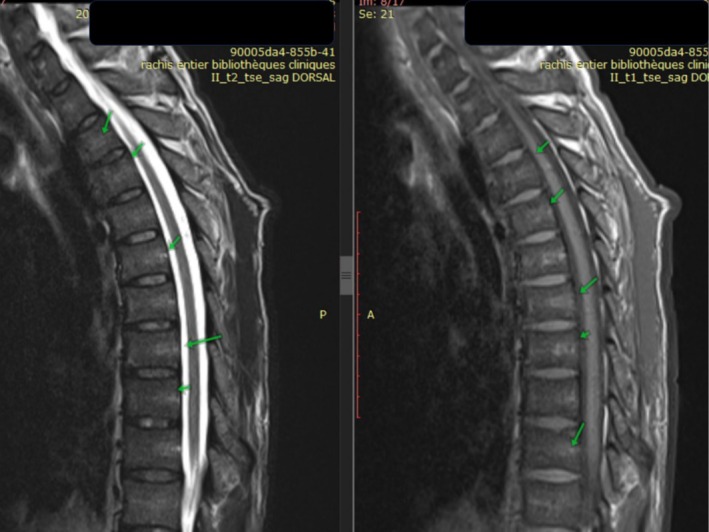
Sagittal T1 and T2‐weighted MRI of the thoracic spine. Extensive band‐like hyperintense signal involving multiple thoracic vertebral bodies (green arrows) with maintained disc height and signal.

**FIGURE 3:**
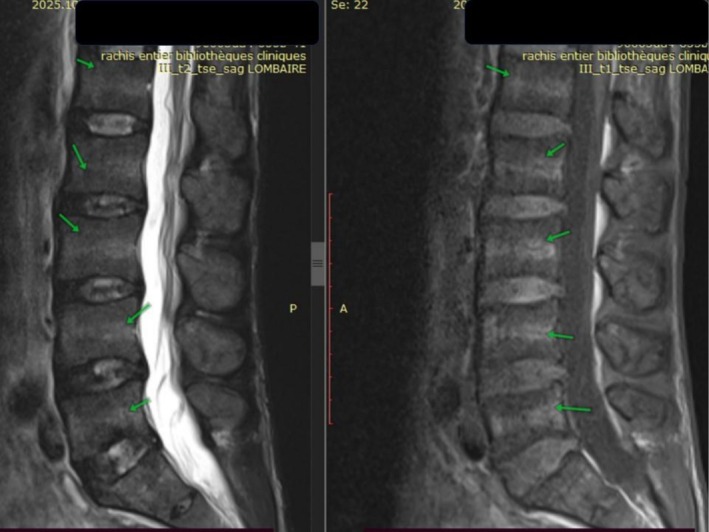
Sagittal T1 and T2‐weighted MRI of the lumbar spine. Diffuse hyperintense signal infiltration of lumbar vertebral bodies (green arrows) demonstrating the characteristic band‐like pattern seen throughout the entire spine.

**FIGURE 4:**
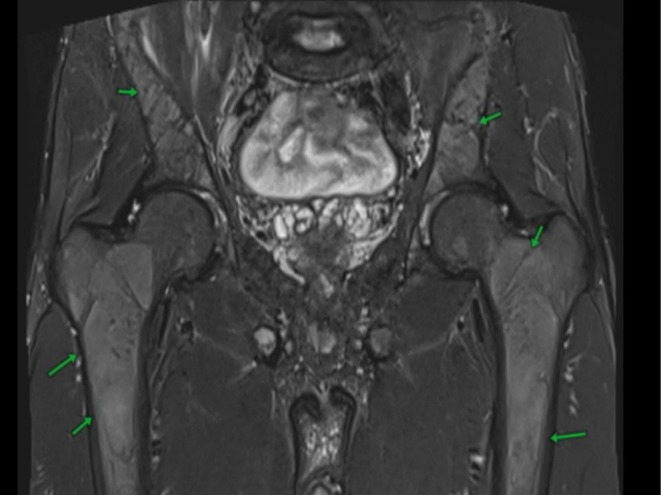
Coronal MRI of the pelvis. Extensive nodular hyperintense lesions involving the sacrum, bilateral iliac wings, and proximal femurs at the cervico‐trochanteric regions (green arrows).

**FIGURE 5:**
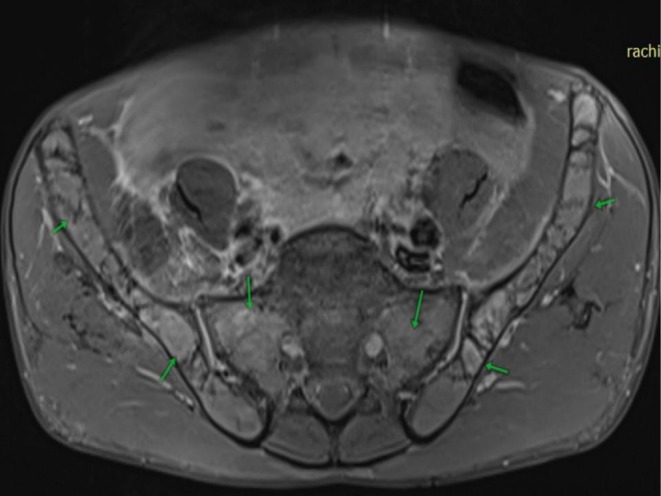
Axial MRI of the pelvis. Axial view demonstrating bilateral symmetric involvement of the proximal femurs with hyperintense nodular lesions at the cervico‐trochanteric regions (green arrows).

### 2.4 Diagnostic Reasoning

In the absence of bone biopsy capability in our setting, we established the diagnosis of probable dialysis‐related amyloidosis based on several compelling arguments. First, the patient had an 8 year history of hemodialysis with low‐flux membranes, representing a well‐established risk factor for β2‐m accumulation. Second, serum β2‐microglobulin was markedly elevated at 22 times the normal upper limit, consistent with inadequate clearance. Third, the imaging pattern was highly suggestive: diffuse bone involvement with preserved disc spaces on MRI, excluding infectious or metastatic processes. Fourth, the polyarticular inflammatory presentation involving large joints corresponds to the known spectrum of DRA manifestations. Finally, the negative immunological markers excluded other inflammatory arthropathies. The moderately elevated PTH with preserved calcium and phosphate levels, in conjunction with normal alkaline phosphatase, argued against severe renal osteodystrophy or secondary hyperparathyroidism as the primary cause of the patient's symptoms.

We ruled out other forms of amyloidosis based on clinical and laboratory grounds. AL amyloidosis was excluded by the absence of monoclonal protein, lack of visceral organ involvement, and the presence of chronic kidney disease predating the bone symptoms. AA amyloidosis was unlikely given the negative inflammatory markers for chronic inflammatory conditions, absence of chronic infections, and the specific osteoarticular distribution of lesions. Hereditary amyloidosis was not supported by the patient's age at presentation, absence of family history, and clear association with dialysis duration.

### 2.5 Therapeutic Intervention

Given the inflammatory component and absence of active gastrointestinal disease, we initiated ambulatory treatment with celecoxib 200 mg once daily for 10 days, followed by as‐needed dosing during painful flares. We counseled the patient regarding the importance of dialysis optimization and discussed the potential benefits of high‐flux membranes or alternative renal replacement strategies, though these options were not immediately available in our resource‐limited setting.

### 2.6 Follow‐Up and Outcomes

At 1 month follow‐up, the patient demonstrated significant clinical improvement. Pain intensity decreased to 1/10 on the visual analog scale, and C‐reactive protein normalized to 2 mg/L. The patient reported improved functional capacity and quality of life. He continued maintenance hemodialysis with ongoing rheumatological monitoring for disease progression.

## 3 Discussion

This case illustrates the clinical challenge of diagnosing dialysis‐related amyloidosis in a resource‐limited setting and highlights several important aspects of this condition in an African patient population.

DRA prevalence has been well documented in developed countries, for example in Japan where the dialysis population grew from 215 patients in 1968 to over 329,000 by 2016 [5]. Reported DRA prevalence rates vary from 5% to 34.1% depending on the anatomical site and diagnostic method used. Tagami et al. found prevalence of 10.6% and 11.1% respectively on radiographic examination, while earlier studies by Fiocchi et al. and Leon et al. reported higher rates of 18% and 19.4% [6, 7].

Duration of dialysis remains the most consistent risk factor for DRA. Koch reported that DRA appears inevitable after 20 years of dialysis, though Hoshino et al. noted decreasing onset risk in recent years due to improved dialysis technology [8, 9].

In contrast, very few DRA cases have been described in sub‐Saharan Africa, likely reflecting limited dialysis access, lower survival rates, scarce diagnostic resources, and underrecognition [10, 11]. Our patient developed symptomatic disease after 8 years of hemodialysis, within the expected timeframe. However, his age at presentation is relatively young compared to Western cohorts. This reflects the distinct demographic profile of African dialysis patients, where chronic kidney disease often results from hypertension and glomerulonephritis in younger individuals [10, 11].

The pathogenesis of DRA centers on β2‐microglobulin accumulation, an 11.8 kDa protein normally cleared by kidneys through glomerular filtration [12]. In end‐stage renal disease, particularly with conventional low‐flux hemodialysis, β2‐m accumulates due to inadequate membrane clearance [12].

Interestingly, clinical outcomes correlate more closely with β2‐m removal rate and clearance than absolute serum levels [12]. Improved β2‐m removal rates associate with lower carpal tunnel syndrome incidence, and the HEMO study demonstrated an inverse correlation between β2‐m clearance and mortality. Advanced techniques using high‐flux membranes or polymethylmethacrylate cartridges enhance β2‐m clearance and improve DRA‐associated joint symptoms [12].

Rather than native β2‐m directly causing disease, misfolded molecules aggregate into amyloid fibrils after 5 to 10 years of dialysis [12]. These fibrils preferentially deposit in osteoarticular tissues where local conditions facilitate formation. Once deposited, β2‐m amyloid triggers an inflammatory response mediated by macrophages and cytokines [12].

Matrix metalloproteinase‐3 (MMP‐3), elevated in hemodialysis patients particularly those with DRA, drives cartilage and bone matrix degradation. Inflammatory cytokines including interleukin‐6 (IL‐6) and tumor necrosis factor‐alpha (TNF‐α) amplify tissue damage [12]. Studies show PMMA cartridges improve removal of MMP‐3 and IL‐6, potentially attenuating disease progression. Alpha‐1‐microglobulin, found in amyloid lesions, also accumulates and may contribute to oxidative stress [12].

The anatomical distribution explains clinical manifestations with carpal tunnel syndrome from flexor retinaculum deposits, destructive spondyloarthropathy from intervertebral disc involvement, and large joint disease from synovial deposition. Our patient's extensive involvement after 8 years of low‐flux dialysis, despite relatively young age, underscores how inadequate β2‐m clearance accelerates disease development.

The presenting symptom of diffuse bone pain in our patient represents an underrecognized manifestation of DRA. While carpal tunnel syndrome, destructive spondyloarthropathy, and bone cysts are well‐documented complications, severe diffuse bone pain as the predominant feature deserves greater clinical attention [13]. Our patient's 2 year history of intermittent inflammatory polyarthralgia affecting multiple large joints initially suggested seronegative spondyloarthropathy or other systemic inflammatory arthropathies. The progression from a mixed mechanical‐inflammatory pattern to predominantly inflammatory characteristics, culminating in severe hyperalgic episodes, highlights the evolving nature of DRA manifestations.

The polyarticular involvement in our case contrasts with more focal presentations typically described. Unlike this incidental radiological discovery, our patient presented with debilitating symptomatic disease requiring urgent rheumatological intervention, emphasizing the clinical spectrum of DRA from asymptomatic radiological findings to severely symptomatic presentations.

The MRI findings proved diagnostic. Band‐like T1 and T2 hyperintense vertebral infiltration with preserved disc spaces represents a highly characteristic pattern for β2‐m amyloid deposition, effectively excluding infectious spondylodiscitis or metastatic disease [14, 15]. The extensive nodular involvement of the sacrum, iliac wings, and proximal femurs represents unusually aggressive disease for an 8 year dialysis duration. The characteristic MRI pattern, while highly suggestive of β2‐m amyloid deposition, cannot establish definitive diagnosis without histological confirmation. Most reported cases demonstrate such extensive skeletal involvement only after 15–20 years of dialysis [16, 17]. This accelerated progression likely reflects prolonged exposure to low‐flux dialysis with inadequate β2‐m clearance, compounded by twice‐weekly rather than thrice‐weekly dialysis frequency.

Our patient's relatively age further distinguishes this case from typical cohorts. Most DRA series report mean ages of 60–70 years, reflecting both the older demographic of dialysis populations in high‐income countries and the prolonged survival required for disease manifestation [5]. The earlier onset of renal failure in our patient, combined with 8 years of suboptimal dialysis, created conditions for accelerated amyloid deposition despite younger age.

The absence of histopathological confirmation through bone biopsy represents the primary limitation of this report, though this reflects the reality of clinical practice in resource‐limited settings where such procedures remain unavailable. The lack of long‐term follow‐up data prevents assessment of disease progression and treatment efficacy beyond the initial 1 month response. We were unable to offer advanced therapeutic interventions such as high‐flux dialysis membranes, hemodiafiltration, or renal transplantation, which limits our ability to comment on optimal disease management. Finally, serum β2‐microglobulin monitoring over time was not performed, precluding correlation between biochemical markers and clinical response.

## 4 Conclusion

This case report highlights that dialysis‐related amyloidosis should be considered in the differential diagnosis of musculoskeletal complaints in long‐term dialysis patients in sub‐Saharan Africa. While we cannot generalize from a single case, our experience suggests that careful clinical and radiological assessment can guide diagnosis when histological confirmation is unavailable. Further case series and prospective studies are needed to better characterize the epidemiology and clinical spectrum of DRA in African populations as dialysis access continues to expand.

## Author Contributions

**Yannick Laurent Tchenadoyo Bayala:** conceptualization, data curation, formal analysis, methodology, project administration, resources, writing – original draft, writing – review and editing. **Mohamed Hanas Mama Sika:** conceptualization, resources, software. **Marina Saley André Hamidou:** conceptualization, data curation. **Issa Ouedraogo:** resources, software. **Rachidatou Kafando:** conceptualization, data curation. **Bakoubassé Aïssata Son:** formal analysis, investigation. **Wendyam Nadège Yameogo:** validation, visualization. **Wendlassida Joëlle Stéphanie Zabsonré/Tiendrébéogo:** writing – original draft, writing – review and editing. **Dieu‐Donné Ouedraogo:** supervision.

## Funding

The authors have nothing to report.

## Consent

Written informed consent was obtained from the patient for publication of the details of their medical case and any accompanying images.

## Data Availability

The data that support the findings of this study are available from the corresponding author upon reasonable request.
